# A conserved motif in the disordered linker of human MLH1 is vital for DNA mismatch repair and its function is diminished by a cancer family mutation

**DOI:** 10.1093/nar/gkad418

**Published:** 2023-05-24

**Authors:** Karla Wolf, Jan Kosinski, Toby J Gibson, Nicole Wesch, Volker Dötsch, Maurizio Genuardi, Emanuela Lucci Cordisco, Stefan Zeuzem, Angela Brieger, Guido Plotz

**Affiliations:** Department of Internal Medicine 1, University Hospital, Goethe University, Frankfurt am Main, 60590, Germany; European Molecular Biology Laboratory (EMBL), Centre for Structural Systems Biology (CSSB), Hamburg, 22607, Germany; European Molecular Biology Laboratory (EMBL), Structural and Computational Biology Unit, Heidelberg, 69117, Germany; Institute of Biophysical Chemistry and Center for Biomolecular Magnetic Resonance, Goethe University, Frankfurt am Main, 60438, Germany; Institute of Biophysical Chemistry and Center for Biomolecular Magnetic Resonance, Goethe University, Frankfurt am Main, 60438, Germany; UOC Genetica Medica, Fondazione Policlinico Universitario A. Gemelli IRCCS, Rome00168, Italy; Dipartimento di Scienze della Vita e di Sanità Pubblica, Università Cattolica del Sacro Cuore, Rome00168, Italy; Department of Internal Medicine 1, University Hospital, Goethe University, Frankfurt am Main, 60590, Germany; Department of Internal Medicine 1, University Hospital, Goethe University, Frankfurt am Main, 60590, Germany; Department of Internal Medicine 1, University Hospital, Goethe University, Frankfurt am Main, 60590, Germany

## Abstract

DNA mismatch repair (MMR) is essential for correction of DNA replication errors. Germline mutations of the human MMR gene MLH1 are the major cause of Lynch syndrome, a heritable cancer predisposition. In the MLH1 protein, a non-conserved, intrinsically disordered region connects two conserved, catalytically active structured domains of MLH1. This region has as yet been regarded as a flexible spacer, and missense alterations in this region have been considered non-pathogenic. However, we have identified and investigated a small motif (ConMot) in this linker which is conserved in eukaryotes. Deletion of the ConMot or scrambling of the motif abolished mismatch repair activity. A mutation from a cancer family within the motif (p.Arg385Pro) also inactivated MMR, suggesting that ConMot alterations can be causative for Lynch syndrome. Intriguingly, the mismatch repair defect of the ConMot variants could be restored by addition of a ConMot peptide containing the deleted sequence. This is the first instance of a DNA mismatch repair defect conferred by a mutation that can be overcome by addition of a small molecule. Based on the experimental data and AlphaFold2 predictions, we suggest that the ConMot may bind close to the C-terminal MLH1-PMS2 endonuclease and modulate its activation during the MMR process.

## Graphical Abstract

**Graphical Abstract:**
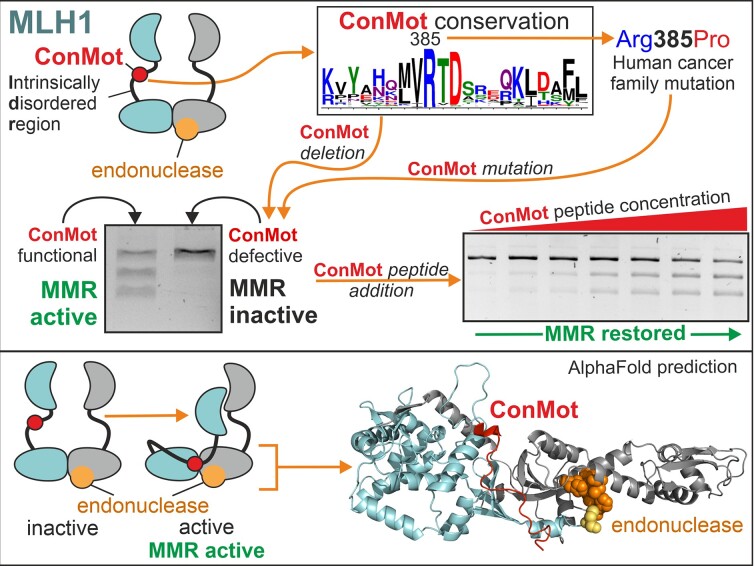

## INTRODUCTION

DNA mismatch repair (MMR) is a highly conserved repair system; it is active predominantly in the correction of base-base mismatches and insertion-deletion loops that occur during replication. It increases replication fidelity by three orders of magnitude (1). Its heterozygous inactivation in the germline causes Lynch syndrome (OMIM #120435), a dominantly heritable cancer predisposition which affects mainly endometrium and colorectum, but also other organs. Lynch syndrome accounts for 2–5% of all colorectal cancer (CRC) cases, but approximately 15% of sporadic CRC patients also show somatic inactivation of MMR, mostly due to hypermethylation of the MLH1 promoter (2).

Two protein dimers are the major components of DNA mismatch repair: both MutS and MutL form homodimers in prokaryotes and heterodimers in eukaryotes. In humans, MSH2-MSH6/3 and MLH1-PMS2 are the major repair proteins. Genetic inactivation of MLH1, MSH2, MSH6 and PMS2 underlies Lynch syndrome (3–6).

MutS proteins are ATPases and versatile detectors of base mismatches and insertion-deletion loops in the DNA duplex (5,7–10). Their mode of mismatch detection has been studied in several crystal structures and involves kinking of the DNA duplex at the mismatched site (for review, see (11)). Minimal MMR reactions have been reconstituted *in vitro* from purified components and, in case of the human system, rely on MutSα, MutLα and some additional factors (RPA, RFC, PCNA, exonuclease I and DNA polymerase) (12–14). However, the mechanism of the repair reaction is still under discussion (15–17). It is difficult to assess experimentally since both protein dimers can assume a multitude of conformational states, depending on the loading of their ATP binding sites and on interactions with homoduplex DNA, heteroduplex DNA or with each other (18–21). Moreover, MutS proteins may utilize different modes of movement on DNA (for mismatch search and for repair initiation) (for review, see (22)). The repair reaction is initiated by mismatch recognition, which authorizes binding of ATP by MutS (21,23). This induces a comprehensive conformational transition that has been found to transform MutS proteins into stably sliding DNA clamps, and sliding of MutS proteins as well as of MutS-MutL complexes is considered to be an important intermediate of the DNA mismatch repair reaction (24–28). Alternatively, it has also been suggested that MutL directs MutS to remain stationary in the vicinity of the mismatch after ATP binding, suggesting that repair is initiated by mispair-bound MutS-MutL complexes (29,30). ATP binding allows interaction of MutS with MutL dimers via their N-terminal domain (19,21,31,32). Before initiating DNA strand removal, MMR has to identify the newly replicated (and therefore erroneous) strand. While hemi-methylated GATC sites are used in some gammaproteobacteria for this task (33), interaction with PCNA at the replication fork is considered to allow strand discrimination in other organisms (34–38). After strand identification, the endonucleolytic activity of MutL proteins is activated (39,40). This activation is PCNA-dependent and introduces nicks in the wrongly replicated DNA strand in vicinity to the mismatch, possibly through direct interactions of MutL N-terminal domains (NTD) and C-terminal domains (CTD) (41–46). The nicks are introduced on both 5’- and 3’-sides of the mismatch in the newly replicated strand to facilitate its digestion (46–49).

Therefore, MutL proteins are mediators between mismatch recognition and removal of the faulty DNA strand. Their precise mode of function is still under discussion. MutL proteins contain ATPase activities in their structured NTD (50). Furthermore, their structured CTDs confer constitutive dimerization (51,52). ATP binding has been found to cause transient dimerization of the NTD (53) and conformational condensation of the protein (44). MutL proteins possess DNA binding capability (49,54–57). They have been observed to be able to move on DNA duplexes and even traverse obstacles like nucleosomes (27,58,59), presumably by formation of a DNA ring using their flexible linkers and the dimerization of the N-terminal domains (11). Additionally, there is evidence that MutL proteins cooperatively bind DNA and can form active polymers on DNA (60,54).

Eukaryotic MutL proteins interact with DNA and mismatch repair components like MutS proteins and PCNA (31,32,54,61–64), but also with further proteins, conferring effects on proliferation, metastasis and apoptosis control (65–69), therefore they can be regarded as versatile adaptors for diverse purposes exceeding their immediate role in mismatch repair.

While the catalytic activities of both structured domains of MutL (NTD and CTD) have been investigated, it is unclear how both domains communicate to facilitate the repair reaction and which role the MutL linkers play. The linkers represent intrinsically disordered regions (IDR) (70). Although unstructured, such regions occur in about one third of eukaryotic proteins, and they often fulfil specific functions, frequently involving protein interactions, either within the same or with other proteins (71–73). Small motifs or modules located within these IDRs may facilitate such tasks (74). The linker regions in MLH1 and PMS2 show no general evolutionary conservation in sequence and have significant differences in length, being 100–300 amino acids long, in case of the PMS2 homologue MLH3 even 800 amino acids. Small coding variations in these regions found in patients are considered non-pathogenic because practically all pathogenic variants have as yet been observed in the structured N- and C-terminal domains (15). Although they are flexible and of significant length, the linkers are required for repair since proteolytic cleavage of the linker *in vitro* reduces MutL DNA binding capacity, and cleavage *in vivo* abrogates MMR (75). Deletions in the yeast MLH1 linker conferred effects on MMR capacity, DNA-stimulated ATPase activity, movement on DNA and nicking (76). It was concluded that the linkers are relevant for completing the ATPase cycle and therefore for both movements on DNA and efficient endonuclease activation. A recent study using ‘handcuffing’ of the linkers via insertion of rapamycin-dependent FRB and FKBP dimerization domains in specific positions of the MLH1 and PMS2 linkers showed that inter-dimer handcuffing close the C-terminal dimerization domain caused none or weak effects, while N-terminal handcuffing conferred decreased MMR activity and DNA binding (77). However, ATPase- and endonuclease activities were increased, suggesting an inappropriate activation of MutLα. Handcuffing the MLH1 linker with itself displayed the most pronounced MMR defect: while no effect on ATPase activity was detectable, DNA binding and endonuclease activity were reduced, suggesting that the MLH1 linker may be important for conformational rearrangements bringing the DNA strand to the endonuclease site.

We have investigated the human MLH1 linker region and identified a small motif (subsequently termed ConMot) that, in contrast to the rest of the linker, displays a high degree of conservation exclusively in eukaryotes. Genetic variants of unclear clinical significance have been reported in this motif in cancer patients. We have analyzed the role of the ConMot and the variants located therein concerning their effects on protein expression and mismatch repair activity.

## MATERIALS AND METHODS

### Cell culture, reagents, vectors and variant synthesis

HEK293 and HEK293T cells were purchased at DSMZ German Collection of Microorganisms and Cell Culture (Braunschweig, Germany) and maintained in D-MEM containing 10% FCS and antibiotics/antimycotics.

The pcDNA3 expression vector containing the entire open reading frame of human MLH1 was a gift of Dr Hong Zhang (Huntsman Cancer Institute, University of Utah, Salt Lake City, UT, USA). The pSG5 expression vector containing full-length human PMS2 cDNA was provided by Dr Bert Vogelstein (Johns Hopkins Oncology Center, Baltimore, MD, USA). Amino-acid positions in MLH1 refer to the 756 aminoacid reference MLH1 sequence (NP_000240.1).

Sequence variants of the MLH1 expression vector were generated using the Q5 site directed mutagenesis system (New England Biolabs, Ipswich, MA, USA) with the appropriate primers (Supplementary Table S1).

Four peptides were used for the complementation assays: a 32-mer peptide containing the sequence of the MLH1-ConMot (ConMot-32, SGSSDKVYAHQMVRTDSREQKLDAFLQPLSK), a 32-mer peptide with a scrambled sequence of the MLH1-ConMot (Scramble-32, SGSSDMVQFEKLKDRRSQDATYVAHLQPLSKP), a 20-mer peptide which contains the conserved sequence of the ConMot (ConMot-20, KVYAHQMVRTDSREQKLDAF). These three peptides were synthesized by GenScript (Leiden, Netherlands). Furthermore, another 32-mer control peptide (H3) with the Histone H3-N-terminal sequence (ARTKQTARKSTGGKAPRKQLATKAARKSAPAT) purchased from EpiCypher, Durham, USA, was used.

### Transfection, protein extraction and expression analysis

MLH1-deficient HEK293T cells (78) were transiently transfected with 5 mg of vector DNA and 20 mL of polyethyleneimine (1 mg/ml, ‘Max’ linear, 40 kDa, Polysciences,Warrington, PA) and extracted as described previously (79). The extracts were analyzed by SDS-PAGE and immunoblotting (using anti-MLH1, G168–728, BD Biosciences, and anti-PMS2, E-19, and anti-beta-Actin, C2, from Santa Cruz Biotechnologies). Chemiluminescence signals (Immobilon, Millipore) were detected in an LAS-4000 mini camera (Fuji) and quantified using Multi Gauge v3.2.

For determining MLH1 concentration, pure MutLα (MLH1-PMS2) was used, which was a kind gift of Josef Jiricny, Zürich, Switzerland (80).

### Mismatch repair assay

Mismatch repair activity was assessed as described in detail before (79,81) (Supplementary Figure S3). In short, 50 μg of nuclear extract of HEK293T cells, which do not express MLH1-PMS2 due to a hypermethylation of the MLH1 promoter (78), was supplemented with 5 μg native extract of HEK293T cells co-transfected with plasmids encoding MLH1 and PMS2; the MLH1 plasmid either encoded the wildtype sequence or the variant to be investigated. Mismatched DNA plasmid substrate (Supplementary Figure S3) prepared according to previously published procedures (81–83) was added, and reactions in a final volume of 25 μl were incubated at 37°C for 15 min. Repair efficiency was scored by purifying the mismatched DNA plasmid from the reaction and assessing the fraction of homoduplex sequence by restriction digestion of the mismatched site using EcoRV. The substrate was additionally linearized with AseI.

For complementation assays with peptide, the indicated peptide(s) were used at stock concentrations of 50 or 500 μM in HEPES–KOH 20 mM pH 7.6 and supplemented to the MMR reactions.

### Circular dichroism (CD) and melting analysis of ConMot peptide

For the CD analysis, 33 μM ConMot peptide was used in 20 mM Na-phosphate buffer pH 7.2, 20°C. Three scans were performed with 190–300 nm wavelength, using a 1 mm light path. A blank was measured with buffer. For the melting analysis, 33 μM ConMot peptide was used in 20 mM Na-phosphate buffer pH 7.2. Detection was at 222 nm, temperature was scanned from 25–95°C. A blank with buffer only was subtracted.

### Bioinformatic analyses

Sequences for alignments were retrieved using BLAST with the human MLH1 protein sequence (NM_000240) as query. Retrieved sequences were specified to organisms’ classes or kingdoms in question by restricting the search on certain TaxIDs. The resulting hits were manually curated according to established procedures (84). Only one MLH1 sequence per organism was retained, removal of incomplete sequences, and verification that the retrieved sequence corresponds to an MLH1 (and not PMS2 or other) protein, which can easily be identified by the highly conserved, C-terminal FERC sequence of eukaryotic MLH1 proteins (48,85). By that procedure, 567 sequences from animals, 348 from fungi, 117 from embryophyta could be identified, while few sequences were available from other eukaryotic kingdoms (3 from red algea, 9 from green algea, 8 from amoebozoae, 1 apusozoa and 2 choangoflagellata). Sequences were handled in JalView (86). Alignments were performed with Muscle (87). Conservation scores were calculated with these alignments using ConSeq (88). Conservation images of motifs were created using WebLogo 3 (89).

The model of MLH1-PMS2 complex was built using AlphaFold (90) version 2.2.2 with all options set to default except the max_recycles parameter which was increased from 3 to 12 maximise model accuracy. AlphaFold was run using AlphaPulldown pipeline (91).

Peptide folding predictions were performed using PEP-FOLD3 (92).

## RESULTS

### Eukaryotes have a conserved motif within the intrinsically disordered MLH1 linker region

**Figure 1.**
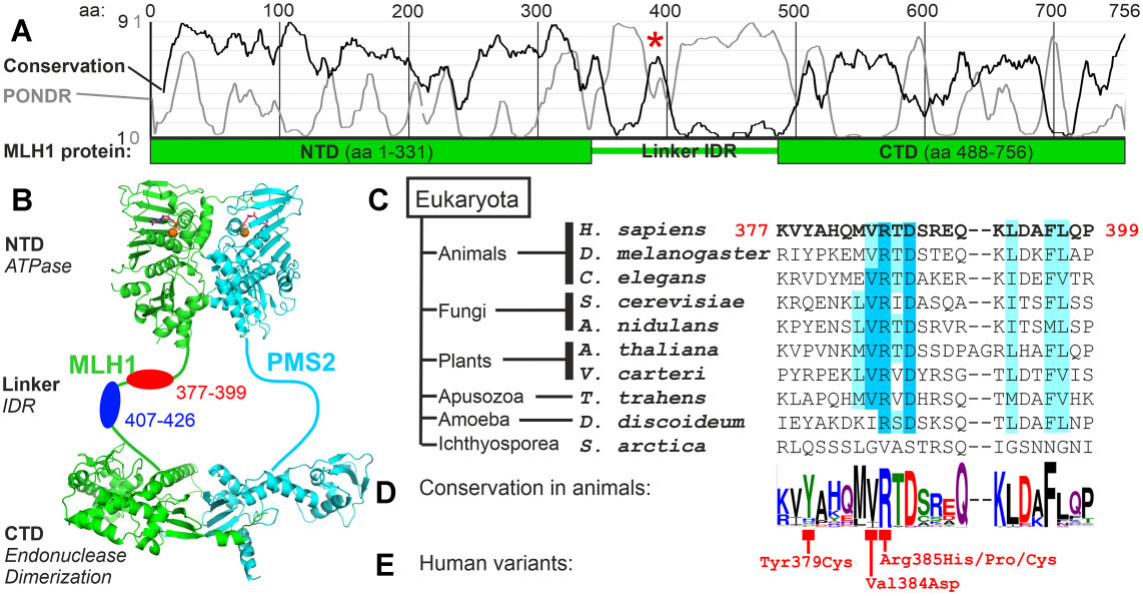
Location, conservation and human variation of a conserved MLH1 linker motif. (**A**) Conservation scores of the MLH1 protein sequence were calculated using ConSeq, resulting in no conservation (score 1) to very high conservation (score 9). The PONDR score was calculated using the human MLH1 sequence; scores above 0.5 are associated with intrinsically disordered regions (IDRs). The linker region displays low conservation and high PONDR scores except for a small area (marked by an asterisk). (**B**) Overview of the MutLα heterodimer consisting of MLH1 and PMS2. Both contain structured, N-terminal ATPase domains (NTD) capable of transient dimerization and structured C-terminal endonuclease domains (CTD) conferring constitutive dimerization. NTD and CTD are connected by a linker IDR. The conserved area (aa 377–396) is located in this linker (red). A control region deleted for subsequently described experiments is also shown (blue). (**C**) Conservation of the conserved motif in organisms of different eukaryotic kingdoms. Dark blue shading represents high conservation, light blue shading weaker conservation. (**D**) WebLogo presentation of conservation in animals. Degree of conservation is represented by letter size, variety of residues in the respective position is given by letters of different sizes. (**E**) Human variants have been observed in the conserved area and are annotated in the WebLogo presentation.

MutL proteins, including human MLH1 and PMS2, possess two structured, highly conserved functional domains: the ATPase function is located in the N-terminus (NTD), while an endonucleolytic activity is located in the C-terminus (CTD) of PMS2 (Figure 1A and B). The CTDs also confer a constitutive dimerization of both proteins (51,52), while the N-terminal ATPase domain can transiently dimerize, controlled by the binding and hydrolysis of ATP (53).

Both structured domains are connected by a non-conserved linker of variable length, which has been suggested to contribute to DNA binding (75). This sequence represents an intrinsically disordered region (IDR) of the protein, as is illustrated by a PONDR analysis (Figure 1A) (93). Consistent with the low sequence and length conservation of this IDR, small coding alterations in this region that have been identified in humans are usually considered non-pathogenic (15).

Depending on the composition of sequences analyzed in MutL alignments, a small conserved area becomes evident within the disordered linker region (Figure 1A). Along with increased conservation, the PONDR score is reduced, suggesting that this area may fold into a secondary structure (Figure 1A). A comprehensive alignment analysis demonstrated that the peak of conservation within the linker corresponds to a highly conserved MVRTD motif found exclusively in MLH1 proteins of the eukaryotic domain, which is embedded in a less conserved motif embracing 22–24 amino acids (Figure 1C and D). Kingdom-specific alignments show that these flanking sequences comprise residues highly conserved within their respective eukaryotic kingdom but showing some variation between kingdoms (Supplementary Figure S1).

The strong sequence conservation suggests a biological significance of this motif which is, for ease of reading flow, termed ConMot subsequently. Notably, within the ConMot sequence, human variants have been identified in cancer patients (Figure 1E) (94).

Further sequence evaluation demonstrated that not only a reduction in PONDR score is predicted for the ConMot, but structure prediction algorithms suggest an elevated propensity to form a secondary structure. The peptide folding algorithm PEP-FOLD3 predicts a potential α-helical structure in two short sequences of the human ConMot, interrupted by the highly conserved MVRTD motif (Supplementary Figure S2A). Therefore, the sequence may provide a potential for folding into a secondary structure (Supplementary Figure S2B). We have analyzed a 32-mer peptide containing the human ConMot sequence by circular dichroism and melting analyses (Supplementary Figure 2C and D). Neither investigation provided evidence for secondary structure formation of the ConMot peptide. However, the investigated 32-mer peptide is rather short for forming stable secondary structures and may lack protein or DNA interactions that under biological conditions would confer secondary structure stabilization.

### Deletion and scrambling of the ConMot and one human variant abolish DNA mismatch repair

A prominent task of MLH1 is its contribution to the DNA mismatch repair reaction. In order to assess the relevance of the ConMot for MLH1 function, we therefore introduced artificial and human variants in MLH1, expressed the MLH1-PMS2 heterodimers in MLH1-deficient HEK293T-cells and investigated their capacity to repair a G-T mismatch in a test plasmid.

We constructed a deletion variant (ΔConMot) in which the conserved ConMot sequence comprising 20 residues was removed (red in Figure 1B). For comparing if the shortening of the MLH1 linker region can disturb mismatch repair by itself, we generated a deletion-comparison variant in which a non-conserved fraction of the linker comprising also 20 residues was removed (ΔCompare) (blue in Figure 1B). For assessing the relevance of the central conserved motif sequence, a scrambled variant was investigated, in which the MVRTD sequence was substituted by the sequence DTMVR.

**Figure 2.**
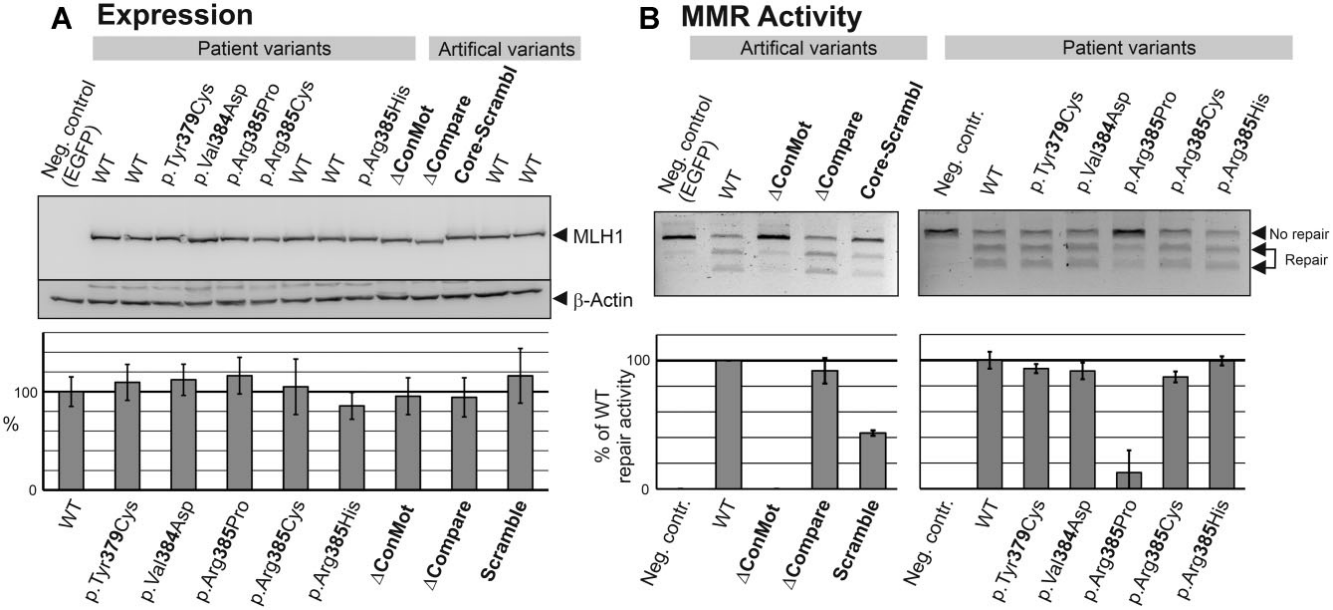
Expression and DNA mismatch repair activity of human and artificial ConMot variants. Expression vectors encoding either WT or variant MLH1 and PMS2 were co-transfected in HEK293T cells which do not express endogenous MLH1-PMS2. As a negative control, one sample was only transfected with an EGFP. After 48 h, native whole cell protein extracts were prepared. Expression was assessed by SDS-PAGE and western blotting. (**A**) Representative western blot of MLH1 wildtype and variant expressions. Average expression levels of MLH1 of > 5 independent transfections were determined and are given in the bar diagram below. (**B**) The extracts of the transfections were used to assess the DNA mismatch repair activities of the variant MLH1 proteins in comparison with wildtype (WT) MLH1-PMS2 as detailed in Materials and Methods and as shown in Supplementary Figure S3. The EGFP-transfected extract sample served as negative control. Representative agarose gels are shown in the top panel. The bottom panel shows average repair values of >3 independent experiments.

Wildtype and variant MLH1-PMS2 proteins were well expressed (Figure 2A). Although most human MLH1 missense variants confer functional defects as well as pathogenicity by destabilizing the MLH1 protein (79), neither the human variants nor the artificial variants in the linker region investigated here had any effect on expression, suggesting that even gross alterations in the linker region do not significantly destabilize the MLH1 protein. This is consistent with the absence of large secondary structures in the linker region, and also suggests that potential interactions of the ConMot with the MLH1 NTD or CTD regions do not significantly contribute to protein stability either. The shortening conferred by the deletion variants was visible by corresponding size shifts (Figure 2A).

We next tested the DNA mismatch repair capacity of all variants by assessing the restoration of a mismatched restriction site in a test plasmid (Supplementary Figure S3). This demonstrated that deletion of the ConMot from MLH1 fully abrogated mismatch repair (ΔConMot), while the more C-terminally located deletion of identical size (ΔCompare) had no detectable effect on repair activity (Figure 2B, left panel). The scrambling of the highly conserved MVRTD motif also significantly reduced repair capacity.

The applied MMR assay procedure is not only suitable for research purposes but can also be applied for pathogenicity clarification of human variants (79,81,95). We therefore tested human variants of the ConMot (Figure 1E). Of these, the proline substitution of Arg385 located within the MVRTD motif displayed a loss of mismatch repair activity (Figure 2B, right panel). The catalytic activities of other substitutions, including a cysteine and a histidine substitution of the same residue, were similar as the wildtype sequence.

**Figure 3.**
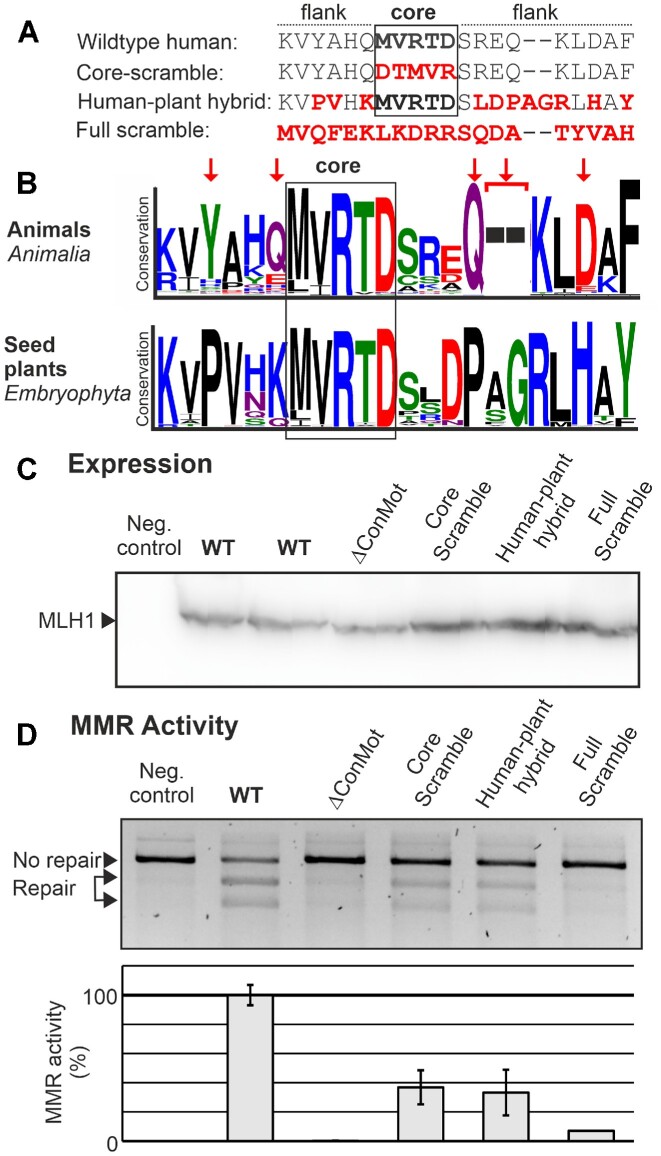
Analysis of the relevance of ConMot core and surrounding sequences. MLH1 proteins containing either the wildtype human ConMot motif sequence or variants were directly compared concerning expression and MMR activity. (**A**) Comparison of the ConMot variants used: the core-scramble variant sequence, the full-scramble variant sequence and an animal-plant hybrid that contains the ConMot consensus sequence of plants as it exists, for example, in walnut and mungo bean (*Vigna radiata* and *Juglans regia*). (**B**) Comparison of conservation in the animal versus plant ConMot in WebLogo representation. Specific differences in conservation within the sequences flanking the core MVRTD sequence are marked by red arrows above the animal sequence. (**C**) Western blot of the expression of the different MLH1-ConMot wildtype (WT) and the variants shown in A after transfection in HEK293T cells; negative control: transfection of EGFP. (**D**) MMR activities were assessed in direct comparison to negative control and wildtype human MLH1 as in Figure 2B. Average activities and standard deviations were calculated from >3 independent experiments.

The core MVRTD motif is highly conserved over the complete eukaryotic domain, while the flanking sequences are also conserved, albeit in a kingdom-specific manner (Supplementary Figure S1). This may suggest that the core motif may be invariant for mechanistical reasons, while the flanking sequence may fulfil other functions that have allowed alternative sequences to evolve. To directly compare the contribution of core sequence versus flanking sequences, we used the core-scramble variant and a hybrid variant with a plant ConMot as it is, for example, present in walnut and mungo bean. Exclusively in the plant kingdom, a two-residue (alanine-glycine) insertion in the ConMot flanking sequence is common (Supplementary Figure S1), and, while conservation is high in plants, the pattern of conservation is different from animals (Figure 3B). Therefore, the in the human-plant hybrid, the ConMot contains a functional flanking sequence, albeit from a different organism.

We compared the core-scramble and human-plant-hybrid variants with the ConMot deletion variant and a full scramble variant. All variants were well expressed (indistinguishable from wildtype) (Figure 3C). The core scramble variant again had approximately 40% activity, which was comparably active as the human-plant hybrid (Figure 3D). This shows that the flanking sequence, although variant in inter-kingdom comparisons, is only fully functional within its species context, and that both core and flanking sequence contribute to a similar degree to the functionality of the ConMot.

### A ConMot peptide restores mismatch repair capacity of ΔConMot

Since the ConMot is localized in the flexible MLH1 linker region, it potentially confers a protein-protein interaction or an interaction with DNA; both could be reconciled with current assumptions on the function of the MLH1 linker.

We performed competition/supplementation tests to gain information on the contribution of the ConMot to the DNA mismatch repair reaction using a 32mer peptide containing the human ConMot sequence.

**Figure 4.**
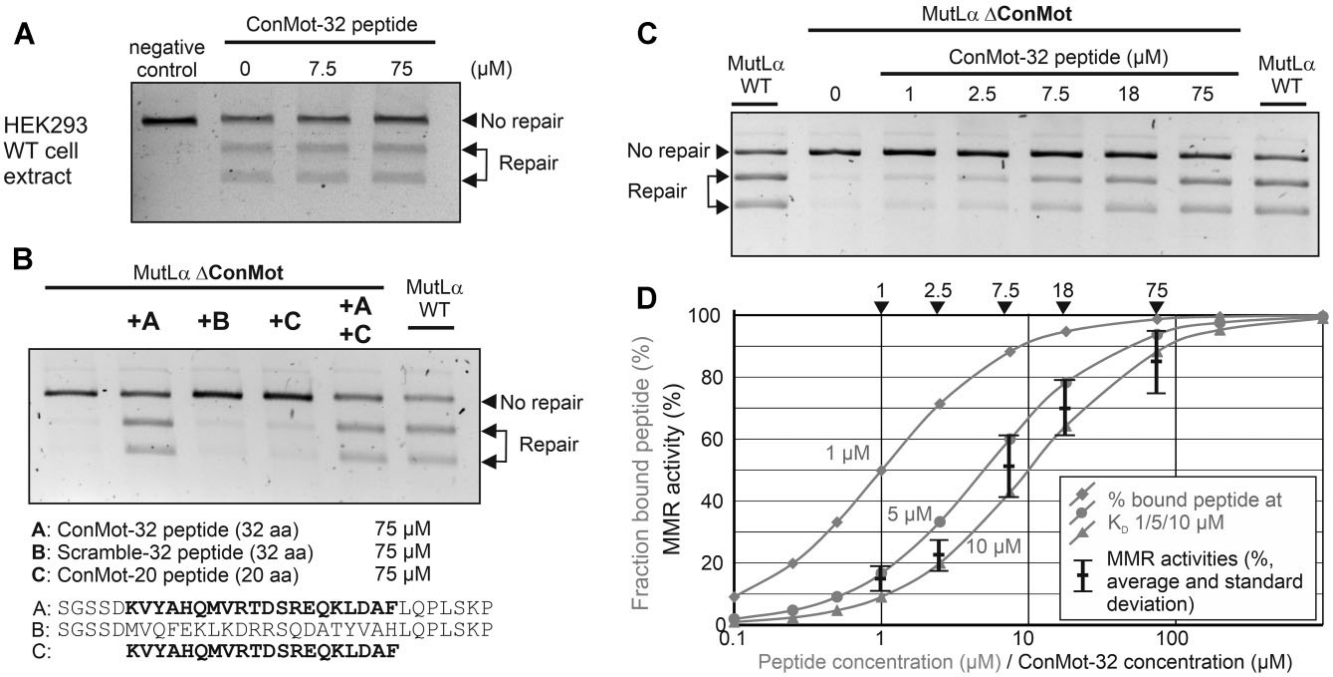
Reaction of DNA mismatch repair to addition of ConMot peptide. (**A**) Nuclear extract of MLH1-proficient HEK293 cells was used for performing the DNA mismatch repair reaction. Four reactions were undertaken, with two reactions supplemented with either 7.5 μM or 75 μM ConMot-32 peptide. A negative control reaction was performed by omitting the 37°C repair incubation. (**B**) MMR reactions were performed with nuclear extract of MLH1-deficient HEK293T cells supplemented with extract of cells transfected either with wildtype MLH1-PMS2 (positive control) or with MLH1-ΔConMot-PMS2. To these MMR reactions, different peptides were added at a concentration of 75 μM: A ConMot-32 peptide (A), a Scramble-32 peptide (B) in which the conserved ConMot sequence is scrambled, and a short ConMot-20 peptide **(C)** that only contains the twenty highly conserved ConMot residues. The peptide sequences are given below the gel image. (**C**) The concentration-dependence of the MMR-activating effect of the ConMot-32 peptide observed in (B) was measured over a concentration range from 1–75 μM. (**D**) Theoretical binding curves of a ligand (peptide) to a protein were calculated for dissociation constants (*K*_D_) of 1, 5 and 10 μM for a protein concentration as present for MLH1-PMS2 in the MMR assay reactions (see also Supplementary Figure S3). Into this diagram, the average MMR activities (and standard deviations) measured in >3 of the experiment exemplarily shown in C were introduced as bars at ConMot-32 peptide concentrations of 1, 2.5, 7.5, 18 and 75 μM.

First, we attempted to interfere with a regular DNA mismatch repair reaction by adding the ConMot peptide to a reaction using extract of MMR-proficient HEK293 cells (Figure 4A). Even 75 μM ConMot peptide did not detectably interfere with the catalytic repair activity of the cell extract, suggesting that the ConMot peptide does not significantly compete with the MLH1-ConMot in a manner causing a defect in mismatch repair.

Second, we used extract of the repair-deficient cell line HEK293T supplemented with either wildtype MutLα (MLH1-PMS2) or the ΔConMot deletion variant, corresponding to experiment shown in Figure 2B. In this experiment, we again added ConMot peptide. Interestingly, the DNA mismatch repair-defect of the ΔConMot variant could be overcome by the addition of exogenous ConMot-peptide (Figure 4B). In contrast, a scrambled control peptide did not restore MMR activity. A smaller peptide (20 aa) containing only the highly conserved ConMot sequence was not active either, suggesting that there is a requirement for additional residues neighboring the ConMot sequence for activity. Since the presence of the 20mer-ConMot did not affect MMR restauration by the 32mer-ConMot, it is likely that target binding, not activation, is compromised in the smaller peptide (Figure 4B).

We investigated the effect of the ConMot peptide more closely by titrating the required peptide concentrations for activation. We determined that the mismatch repair reactions performed in this study contained approximately 280 fmol MLH1 (Supplementary Figure S4A and B), which is in good agreement with previous quantifications of MLH1-PMS2 in cells and extracts (96). An increase in MMR activity became detectable at a ConMot peptide concentration of 1 μM, 50% activation was achieved at 7.5 μM, and almost complete activation at 75 μM (Figure 4C and D). Dissociation constants (*K*_D_) for peptides to proteins are typically in the range of 1–10 μM (97). We compared the measured concentration-dependent MMR activities with theoretical binding curves of (peptide) ligands to (protein) substrates for *K*_D_ values of 1, 5 and 10 μM (Supplementary Figure S4C) at the determined MLH1 concentration. The degree of MMR restoration corresponded very well to the theoretical fraction of peptide binding to a protein for a *K*_D_ of 7.5 μM (Figure 4D). It is therefore plausible that a binding reaction of the ConMot peptide (to a protein) underlies the restauration of MMR activity.

**Figure 5.**
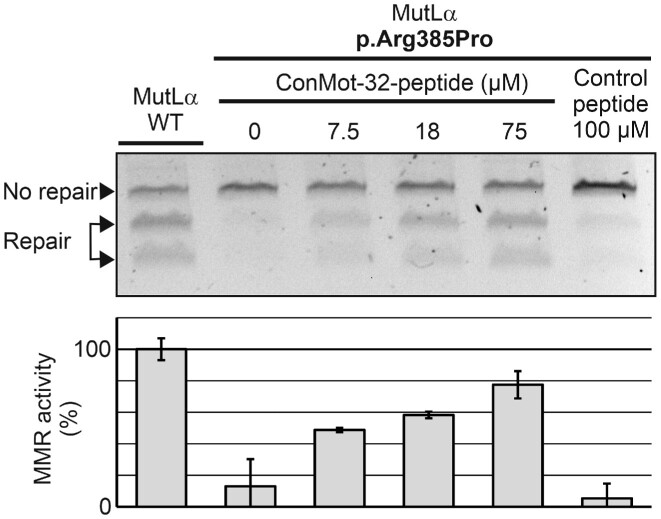
ConMot peptide restores the MMR defect of the p.Arg385Pro patient variant. MMR reactions (Supplementary Figure S3) were performed identically as shown in Figure 4C with nuclear extract of MLH1-PMS2-deficient HEK293T cells, supplemented either with extract of cells transfected with either wildtype (WT) MLH1-PMS2 or with the MLH1 variant p.Arg385Pro and PMS2 as detailed in Materials and Methods. ConMot-32-peptide was added in concentrations of 0, 7.5, 18 and 75 μM. For comparison, a control-peptide (H3) of identical length, but with non-specific sequence, was added at a concentration of 100 μM. Several MMR assays were performed; a representative agarose gel is shown (top), and average MMR activity values and standard deviations of three independent experiments were calculated (bottom).

In an identical way, we analyzed the effects of the ConMot peptide on MMR activity of the defective human MLH1 missense variant p.Arg385Pro (Figure 5). Restoration of MMR activity was again observed, and required identical peptide concentrations as with the ConMot-deletion variant, suggesting that the human p.Arg385Pro variant abolishes the interaction of the MLH1-ConMot with its target binding site, and that addition of ConMot-peptide can replace the function of a mutated ConMot.

### The ConMot variant p.arg385tyr partly restores MMR deficiency of the endonuclease variant p.tyr750arg

**Figure 6.**
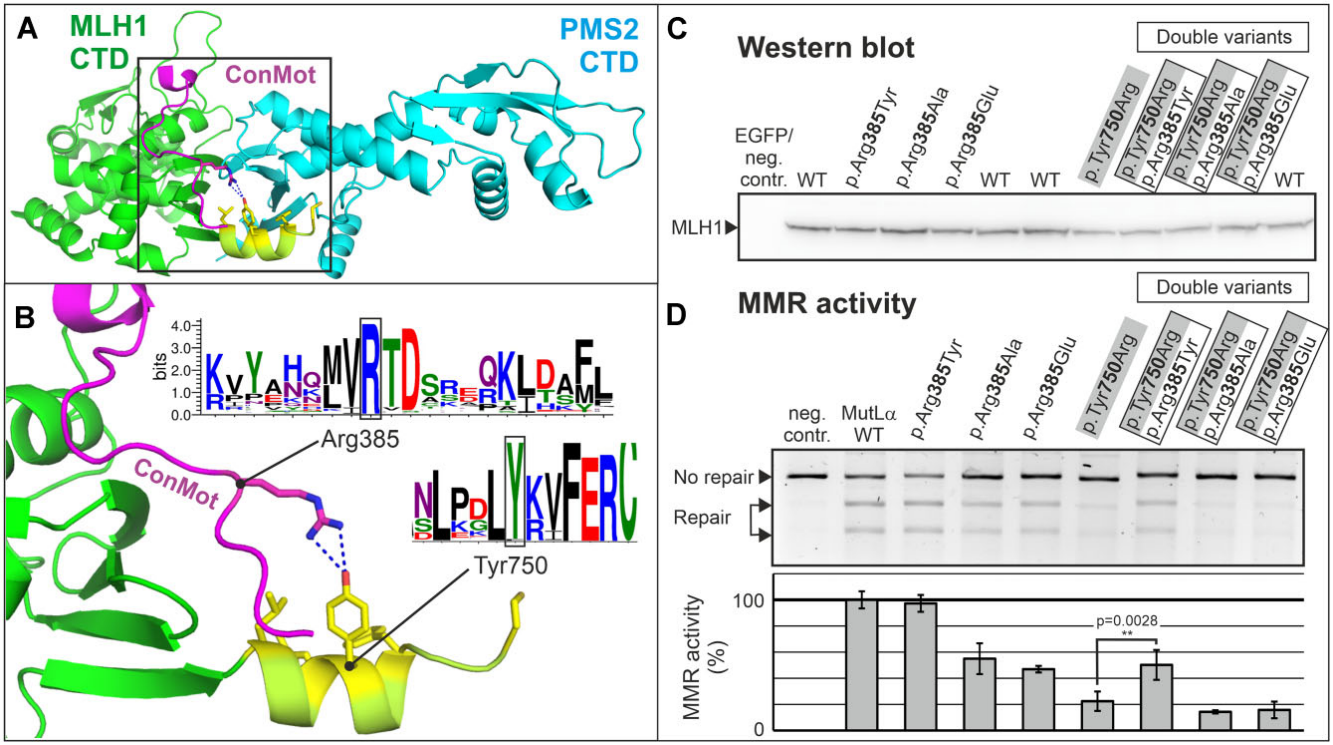
The MMR defect of p.Tyr750Arg is partly compensated by p.Arg385Tyr. (**A**) The AlphaFold2 prediction of the MLH1-PMS2 dimer (MLH1: green; PMS2: cyan) suggests that the ConMot (purple) binds to the CTD of MLH1. The prediction suggests an interaction with the C-terminal helix of MLH1 (light green, hydrophobic residues coloured in yellow), which is involved in Zn^2+^-binding at the PMS2 endonuclease site. (**B**) The side chain of the highly conserved Arg385 is predicted to be in proximity to the side chain of the highly conserved Tyr750 residue. (**C**) MLH1-PMS2-deficient HEK293T cells were co-transfected with the indicated single- and double variants of MLH1 and PMS2. Protein extracts were prepared, separated by SDS-PAGE, blotted and probed for MLH1 protein expression. (**D**) MMR assays were performed to test DNA mismatch repair activity as detailed in Materials and Methods. One representative agarose gel is shown, and average repair values in comparison to wildtype and standard deviations of >3 independent experiments are shown in a bar diagram. A two-sided *t*-test was performed for testing significance of the difference in repair activity of p.Tyr750Arg in comparison to the double variant p.Arg385Tyr + Tyr750Arg.

In our AlphaFold2 model of the MLH1-PMS2 heterodimer, the ConMot is predicted with high confidence to bind to the CTD of MLH1 (Supplementary Figure S5, Figure 6A). The p.Arg385 residue of the ConMot is predicted to be in sufficient proximity to the p.Tyr750 to engage in a side-chain-side-chain interaction (Figure 6B). Both p.Arg385 and p.Tyr750 are highly conserved, with p.Arg385 being the most highly conserved residue of the ConMot (Figure 6B).

We speculated that a putative p.Arg385-p.Tyr750 side chain interaction may be accessible for experimental verification. We performed an exchange of these residues and created the variants p.Arg385Tyr, p.Tyr750Arg and a double variant with interchanged side-chains (p.Arg385Tyr + p.Tyr750Arg). As controls, we generated p.Arg385Ala and p.Arg385Glu. All variant proteins were well expressed (Figure 6C).

While the p.Arg385Tyr substitution was well tolerated in terms of MMR activity, the p.Tyr750Arg alteration caused a comprehensive defect of MMR (Figure 6D). Intriguingly, this defect was partially reverted in the double variant (p.Arg385Tyr + p.Tyr750Arg): repair activity of this double variant was more than twice as high as that of p.Tyr750Arg (it increased from 22.4% to 50.2% on average). This observation was highly reproducible and significant (*P* = 0.0028). This was not the case for the two control alterations of p.Arg385 to alanine and to glutamate: these had no reverting effect on the defect of the p.Tyr750Arg variant, but instead acted additively to result in a more pronounced MMR defect (Figure 6D).

Following the concept of co-evolution, detrimental effects of missense substitutions may potentially be restored by subsequent adaptive substitutions of another residue that is typically in spatial and/or functional contact with the first one (98). Detection of such coupled substitutions in multiple sequence alignments therefore is informative for identifying or confirming direct side chain contacts in three-dimensional structure (99) and is exploited by prediction algorithms as AlphaFold (90).

The expected result of a simultaneous substitution of two independent residues is that a potential defect has at least the same extend as the more deficient single variant. More likely, both may exert an additive effect and result in a more pronounced defect of the combined variant, similar as we have reported before for two small coding variants in MLH1 that conferred an additive defect on protein stability and resulted in a disease phenotype (95). Likewise, in this experiment, the two control variants (p.Arg385Ala and p.Arg385Glu) also reacted in this manner and both additionally aggravated the defect of p.Arg750Tyr.

Therefore, although restauration of repair was incomplete in the p.Arg385Tyr + p.Tyr750Arg double variant, the finding that the second alteration (p.Arg385Tyr) relieved the functional defect of the primary mutation (p.Tyr750Arg) most likely is related to a spatial or functional interaction of both side chains, specifically since both residues were only interchanged. While further investigations are required to confirm and characterize ConMot binding to the MLH1-CTD, this result provides evidence that the interaction of p.Arg385 with p.Tyr750 was correctly predicted by AlphaFold2.

## DISCUSSION

The MutL linker regions have recently attracted increased attention concerning their functional role in the mismatch repair reaction (15,59,76,77). Due to their low degree of sequence and length conservation, human missense alterations are commonly considered innocuous (15). Our investigations demonstrate the existence of a small motif (ConMot) within the human MLH1 linker that is vital for MLH1 function. For a corresponding sequence in yeast *mlh1*, a similar significance has very recently been reported (100). The ConMot motif is exclusively conserved in eukaryotes and comprises 20 amino acids (22 in plants).

### The ConMot sequence and alteration tolerance

The ConMot consists of a central, universally conserved core motif of five amino acids (MVRTD) and of flanking sequence whose conservation is kingdom-specific. While deletion of the ConMot and complete scrambling rendered MLH1 fully deficient in MMR, gross variants of the core motif (core-scramble) and of the flanking sequence (a human chimera containing the flanking sequence of plants) retained approximately 40% catalytic activity. This suggests that its contribution to MMR is not focused on single residues. This is confirmed by the observation that, of all tested missense alterations, only some of the most highly conserved residue Arg385 reduced MMR activity. While three tested substitutions were tolerated (p.Arg385Cys/His/Tyr), three other caused a defect similarly strong as the core-scramble variant (p.Arg385Pro/Ala/Glu). The most pronounced effect was observed for the human patient variant p.Arg385Pro, possibly because the sterically inflexible proline confers a more general strain on target binding by the ConMot than other missense variants and even the core scramble variants. While exact determination of ConMot target binding will be required to explain these results satisfactorily, it seems plausible that ConMot activity primarily depends on its binding to its target, and that this binding has a strongly cooperative nature and is therefore rather tolerant to a number of alterations.

### The potential role of the ConMot in MLH1 function

**Figure 7.**
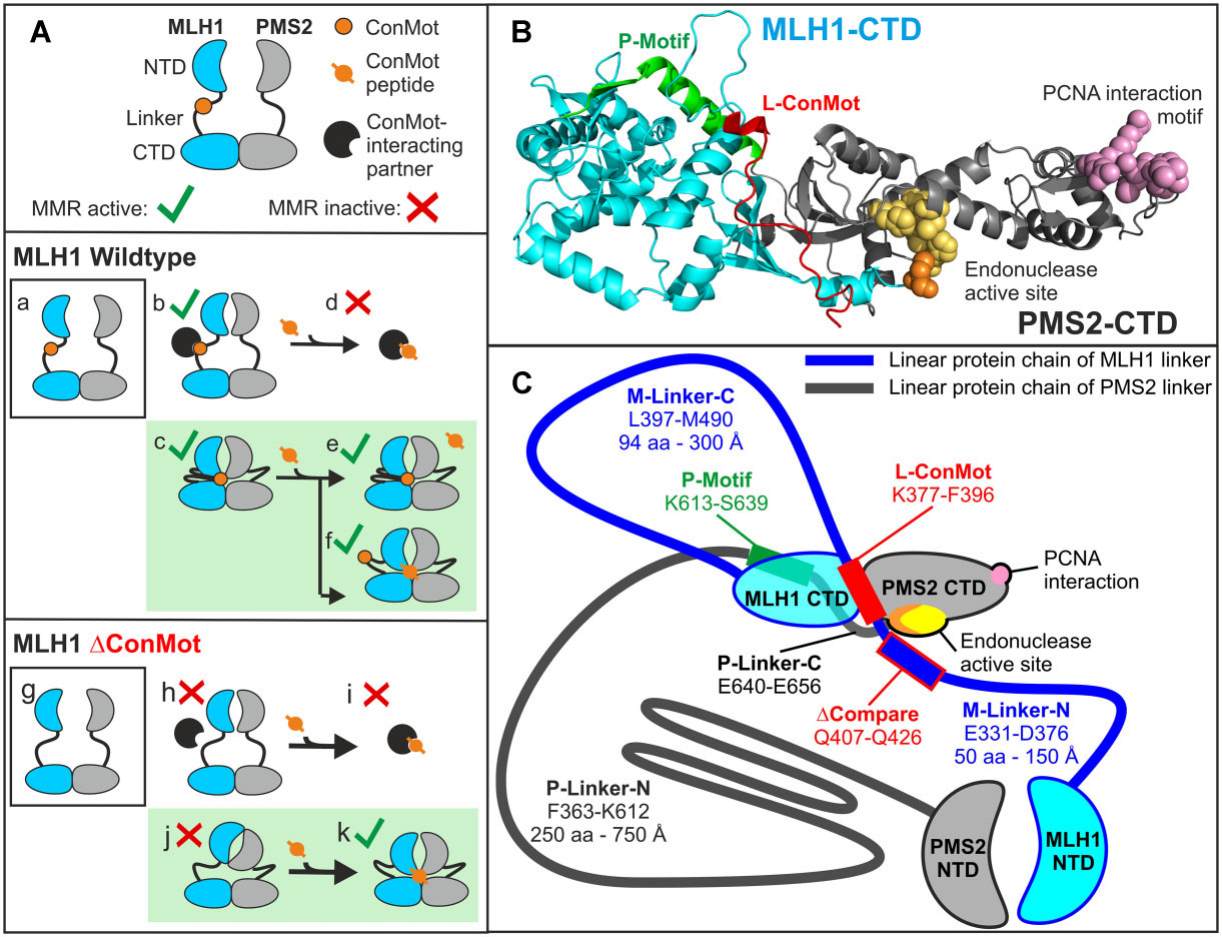
Potential targets and functions of the ConMot motif. (**A**) Potential effects in competition-/supplementation experiments of MLH1-PMS2 proteins with ConMot peptide dependent on the nature of the ConMot-interaction. The scenarios consistent with the experimental findings are shaded green. The MutLα-heterodimer (MLH1-PMS2) is shown with its constitutively dimerized C-terminal domains (CTD) and the ATP-dependently dimerizing N-terminal domains (NTD), and the unstructured linker region in between (**a**). The ConMot is located in the MLH1 linker. The wildtype MutLα heterodimer (WT, a) could either use the ConMot for an interaction with a third partner (**b**), in this case the addition of ConMot peptide is likely to interfere with DNA mismatch repair (**d**). Alternatively, the ConMot may confer an interaction within the dimer (**c**), in this case the addition of exogenous ConMot peptide remains without effect (**e**/**f**). The MLH1-ΔConMot deletion variant (**g**) cannot support the interaction with a putative third partner (protein or DNA) (**h**), resulting in a loss of MMR activity, and addition of exogenous ConMot peptide would not re-establish this interaction or MMR activity (**i**). An interaction of the ConMot within the dimer (**j**) would also be abrogated, leading to a MMR defect, but addition of ConMot peptide could re-establish this interaction and render the complex MMR-proficient (**k**). (**B**) AlphaFold2-prediction of the MLH1-PMS2-dimer: the CTDs and fractions of the linkers binding to the CTD according to this prediction are shown. Residues of the endonuclease active site are shown in spheres (MLH1 Cys756 in orange and PMS2 His703, Glu707, Cys817, Cys 848 and His850 in yellow), residues of the PCNA interaction motif (PMS2 ^723^QKLIIP) in pink spheres. The ConMot (red) is predicted to form a short α-helix and a larger linear fraction and to be bound in full length to the MLH1-CTD close to the dimer interface, with its N-terminal residues touching the last C-terminal MLH1 helix that contributes to the endonuclease active site via Cys756 (orange). The P-motif (green) is composed of a fraction of the PMS2 linker which is predicted to form an α-helix and a short β-sheet that are bound to the MLH1-CTD. (**C**) Schematic representation of size relations of the MLH1-PMS2 structured domains and linkers according to the AlphaFold2-prediction. Lengths and sizes are roughly to scale, corresponding to the MLH1 and PMS2 CTDs which cover approximately 50 Å and 65 Å in width, respectively, and the NTDs which are approximately 65 Å in height. The predicted binding of the ConMot and the P-motif to the MLH1-CTD causes the linkers to form two ring structures: a big one (900 aa) is composed of the N-terminal fractions of both MLH1 linker (M-Linker-N) and PMS2 linker (P-Linker-N) and can open and close by dimerization of the NTDs. The second, smaller one is formed by the C-terminal fraction of the MLH1 linker (M-Linker-N). Both ring structures are potentially big enough to accommodate a DNA double helix.

Since the ConMot is a short motif, it is unlikely to perform any specific activity by its own; rather, it will need to bind a target to perform its function and facilitate MLH1-PMS2 activity. Since the ConMot is situated in the flexible linker, it enjoys some spatial liberty (Figure 7A-a). Our finding that an isolated ConMot peptide can replace the MLH1-ConMot confirms that the exact position of the ConMot within the protein is not of significant importance, suggesting that it moves to its target during the repair process. This is consistent with the observation that the corresponding yeast motif was also active when moved within the yMLH1 linker or even when transferred to the yPMS1 linker (100).

The current results in the peptide complementation experiments allow interesting conclusions concerning the target binding site of the ConMot:

In general, the target may either be another protein or DNA (a third partner, #1, Figure 7A-b), or alternatively, it may perform an interaction within the MLH1-PMS2 dimer (#2) (Figure 7A-c).

We have observed that DNA mismatch repair is not attenuated by addition of even high concentrations of ConMot-peptide (Figure 4A). This finding is supportive of an intramolecular interaction (Figure 7A-c), since formation of a ternary complex with another partner (Figure 7A-b) would be likely impaired in the presence of excess ConMot-peptide by out-competing the MLH1-ConMot for binding to this partner and thereby displacing it from the ternary complex (Figure 7A-d). In contrast, if the MLH1-ConMot is involved in an intramolecular interaction (Figure 7A-c), addition of ConMot-peptide in excess is unlikely to interfere with the DNA repair reaction. Either the ConMot-peptide may be excluded from the complex because it cannot compete with the much more efficient intramolecular interaction of the MLH1-ConMot (Figure 7A-e), or it does displace the MLH1-ConMot without interfering with the repair reaction (which is possible, see below) (Figure 7A-f).

The results observed with the ConMot-deletion variant of MLH1 (ΔConMot, Figure 7A-g) also suggest an interaction of the MLH1-ConMot within the MLH1-PMS2 dimer. In these experiments, it was possible to overcome the MMR-deficiency of the MLH1 ΔConMot variant by addition of exogenous ConMot-peptide. If the MLH1-ConMot were involved in a ternary complex formation, the effect of peptide addition (again) would have been the displacement of the ternary partner, without re-constituting repair activity (Figure 7A-hi). In contrast, the finding that addition of ConMot-peptide restores mismatch repair activity suggests that the ConMot-peptide has re-established an intramolecular or inter-subunit interaction within MLH1-PMS2 (Figure 7A-jk).

It is relevant to note that, in contrast to our results, peptide addition in yeast *inhibited* activity of wildtype yMLH1-yPMS1 (100). However, in these experiments, only a fraction of the complete DNA mismatch repair reaction (non-specific endonuclease activation in the absence of a mismatch) was measured under rather artificial conditions (presence of manganese instead of magnesium), making it possible the observed inhibition arises only in this experimental set-up (see Supplementary Table S2 for a detailed comparison of reaction conditions). Additionally, the use of a shorter peptide (25 aa instead of 32 aa) may have contributed to the conflicting results, since our experiments showed that peptide length is a relevant factor in activation (Figure 4B). Since the experiments described here reflected the behavior of the complete MMR reaction under largely biological conditions and consistently demonstrated non-inhibition of the wildtype reaction and re-constitution of repair in ConMot-mutant variants, we assume that this activating effect better reflects the biological function of the ConMot.

A further aspect corroborating an internal interaction of the ConMot is the observation that the AlphaFold2-prediction, which suggested a binding of the ConMot to the MLH1-CTD with high confidence (Supplementary Figure S5) also predicted a functional interaction of the ConMot residue p.Arg385 with the endonuclease residue p.Tyr750, for which we provided experimental evidence (Figure 6).

In summary, the evidence suggests that the ConMot interacts with a site located within the MLH1-PMS2 dimer (Figure 7B). There, it would be in contact with p.Tyr750 in the C-terminal helix of MLH1 which contributes an essential Zn^2+^ binding Cys residue (Cys765) to the composite MLH1-PMS2 endonuclease site (46,101). Therefore, the ConMot could potentially modulate endonuclease activity, or even contribute catalytically relevant residues, from this position, consistent with the observed MMR defect and with the observation that mutation of the corresponding motif in yeast inactivated endonuclease activity (100). Binding of the ConMot to this site would also be consistent with the observation that ConMot residues were found to be close to the DNA backbone in FeBABE experiments (102), since it is simultaneously located close to the DNA in this proximity to the endonuclease site.

Besides a potential involvement in endonuclease activation, the proposed interaction of the ConMot with the MLH1-CTD would subdivide the central cavity of MLH1-PMS2 that is formed by N-terminal dimerization (Figure 7C). A smaller linker ring of approximately 300 Å circumference would be formed simultaneously with a larger ring comprising the residual fraction of the MLH1-CTD combined with both MLH1- and PMS2-NTD and the major part of the PMS2 linker. This may contribute to a controlled and targeted activity of the endonuclease function to the newly synthesized DNA strand whose identity is communicated by PCNA at the replication fork (38). Alternatively, the ConMot may be involved in formation of MutL active polymers that have been observed to occur on DNA by binding neighboring subunits (60).

Taken together, the ConMot is a conserved, movable, short motif located in an IDR, and likely to confer interaction to a structured protein domain. Therefore, it has features attributed to so-called ‘Short Linear Motifs’ (SLiM), which frequently confer weak and/or transient protein interactions (74,84,103,104).

Besides the interaction of the ConMot with the MLH1-CTD, a helical motif in the PMS2 linker is present in our AlphaFold2-predictions and in previous predictions for yeast and other organisms (100). This P-motif is predicted to bind in the MLH1-CTD (Figure 7B and C). This interaction would tether the most C-terminal portion of the PMS2 linker to the structured MLH1 CTD, causing the PMS2 linker to actually be ‘connected’ to the MLH1-CTD instead of the PMS2-CTD (P-Linker-N in Figure 7C). This has plausibly been suggested to evade sterical problems in interactions during the repair process (100), since the PMS2 linker thereby is at greatest possible distance to the PMS2 PCNA interaction motif (64) and the endonuclease domain (48) (Figure 7C).

### Human variants in the ConMot

As yet, pathogenic small coding variants causing Lynch syndrome have almost exclusively been identified either in the conserved NTD or in the CTD, where they can suppress diverse aspects of MLH1 function (105). In the NTD, they may interfere with the ATPase cycle (50,80) and/or with MLH1-MSH2 interaction (83). In the CTD, they may disturb dimerization (51), frequently affect protein stability (79) and/or may impair endonuclease activity (47,85). In contrast, the MLH1 linker region appeared largely devoid of pathogenic small coding variants (15). Our present data demonstrate that the ConMot represents an exception to this rule.

We investigated several human alterations reported within the ConMot. Of these, the p.Arg385Pro alteration displayed a mismatch repair defect. It is located in the core of the strongly conserved MVRTD motif. The substitution to proline likely introduces a sterical strain that interferes with the ability of the ConMot motif to adopt a proper conformation for target binding. This alteration has originally been identified in a patient with a large adenoma with focal high grade dysplasia and a family history of CRC (family ID R-RM6) (106) (Supplementary Figure S6). Microsatellite instability (MSI) in the tumor tissue is a hallmark of MMR-defective tumors (107), but MSI testing has only been performed in the adenoma tissue of the index patient and therefore was not informative (106). The Arg385Pro allele (rs63750430) has been identified in low frequencies in Asian and European populations (https://gnomad.broadinstitute.org/variant/3-37067243-G-A?dataset = gnomad_r2_1) (108). As with many small coding alterations, insufficient information is available for this alteration, therefore it has remained unclear if this variant is causative for cancer diseases. Correspondingly, it is listed as an ‘variant of uncertain significance’ (class 3) in the clinical reference database for variant classifications (https://www.insight-database.org/classifications/). The MMR defect of this variant confirms that this variant is likely pathogenic and therefore causative for Lynch syndrome in this family by affecting the proper function of the MLH1 ConMot.

While the sample size is too small for a final evaluation, it is interesting to note that the average age at cancer diagnosis is rather high in the family with the p.Arg385Pro variant. We have shown before that incomplete inactivation of MLH1 by missense alterations prompts a milder Lynch syndrome phenotype with lower penetrance and higher average age of cancer onset (79), and a similar observation has recently been published for an MSH2 variant (109). In case of the ConMot, a complete loss of MMR activity was observed only in the deletion and full scramble variants, while other alterations like the ‘Core-Scramble’ and the ‘Animal-plant hybrid’ variants, still retained some activity (Figures 2 and 3). It is therefore possible that small ConMot alterations retain partial activity entailing a milder Lynch syndrome phenotype.

Intriguingly, the DNA mismatch repair defect of the p.Arg385Pro variant could be overcome by addition of ConMot peptide. To our knowledge, this is the first example of restoration of mismatch repair activity to a MMR-deficient human MLH1 mutant by addition of a small compound.

In contrast to p.Arg385Pro, conservative substitutions of this residue to histidine and cysteine (p.Arg385His and p.Arg385Cys) did not affect catalytic activity. For neither substitution, pathogenicity classifications exist due to a lack of clinical data. It is interesting to note that homologous mutations in yeast of both substitutions displayed defective MMR (100). This may reflect different sensitivity of the motif to substitutions in the yeast and the human system.

The p.Tyr379Cys variant has been clinically classified as ‘likely not pathogenic’ (class 2) based on co-segregation data (https://www.insight-database.org/classifications/). Consistent with this, we did not find a catalytic defect for this variant. Although tyrosine 379 is a conserved residue of the ConMot, the substitution is rather conservative, suggesting that the ConMot-target interaction is not significantly disturbed.

Although the p.Val384Asp variant is a non-conservative exchange of a highly conserved, small lipophilic residue, there was no detectable impairment of MMR. Consistent with this experimental result, this alteration is a polymorphism (rs63750447, up to 2.7% of controls) in East Asia, speaking against a causative role in cancer; on the other hand, it is the most prevalent somatic alteration reported in MLH1 in different tumor entities (110). According to our data, the variant is unlikely to be causative for Lynch syndrome; further investigations may show if a causative association with other diseases exists.

In summary, we have described identification and preliminary characterization of a conserved eukaryotic protein motif located in the otherwise unstructured, unconserved linker region of MLH1 proteins. This motif is indispensable for human mismatch repair and most likely performs an intramolecular interaction with the CTD of MLH1 during the repair process. While showing a certain degree of tolerance to substitutions, we have shown that variants in the ConMot can disable mismatch repair and therefore can be causative for Lynch syndrome.

## DATA AVAILABILITY

The data underlying this article are available in the article and in its online supplementary material.

## Supplementary Material

gkad418_Supplemental_File
