# Supplementary material for: A conserved motif in the disordered linker of human MLH1 is vital for DNA mismatch repair and its function is diminished by a cancer family mutation
Source: Nucleic Acids Res. 2023 May 24;51(12):6307–20. doi: 10.1093/nar/gkad418 (PMC10325900; doi:10.1093/nar/gkad418)
Supplement: gkad418_Supplemental_File [file gkad418_supplemental_file.pdf]

SUPPLEMENTARY DATA

A conserved motif

in the disordered linker of human MLH1

is vital for DNA mismatch repair and

its function is diminished by a cancer family mutation.

Karla Wolf[1]
Jan Kosinski[2]
Toby J. Gibson[3]
Nicole Wesch[4]
Volker Dötsch[4]
Maurizio Genuardi[5]
Emanuela Lucci Cordisco[6]
Stefan Zeuzem[1]
Angela Brieger[1]
Guido Plotz[1]

[1] Department of Internal Medicine 1, University Hospital, Goethe University, Frankfurt am Main, Germany
[2] European Molecular Biology Laboratory (EMBL), Centre for Structural Systems Biology (CSSB), Hamburg, Germany;
[3] European Molecular Biology Laboratory (EMBL), Structural and Computational Biology Unit, Heidelberg, Germany
[4] Institute of Biophysical Chemistry and Center for Biomolecular Magnetic Resonance, Goethe University, Frankfurt am Main, Germany
[5] UOC Genetica Medica, Fondazione Policlinico Universitario A. Gemelli IRCCS, Rome, Italy
[6] Dipartimento di Scienze della Vita e di Sanità Pubblica, Università Cattolica del Sacro Cuore, Rome, Italy

Supplementary Table 1

**Variant name/protein description (NP_000240.1)**	**Variant cDNA description (NM_000249.4)**	**Site-directed mutagenesis primer sequence F**	**Site-directed mutagenesis primer sequence R**
p.Tyr379Cys	c.1136A>G	GATAAGGTCTgTGCCCACCAG	ACTACTTCCAGAAGTAGAAGAC
p.Val384Asp (rs63750447)	c.1151T>A	CACCAGATGGaTCGTACAGATTC	GGCATAGACCTTATCACTAC
p.Arg385Cys	c.1153C>T	CCAGATGGTTtGTACAGATTC	TGGGCATAGACCTTATCAC
p.Arg385Pro	c.1154G>C	CAGATGGTTCcTACAGATTCCC	GTGGGCATAGACCTTATC
p.Arg385His	c.1154G>A	CAGATGGTTCaTACAGATTCC	GTGGGCATAGACCTTATC
Deletion ConMot (Δ**ConMot**) (p.Lys377_Phe396del)	c.1129_1188del	CTGCAGCCTCTGAGCAAA	ATCACTACTTCCAGAAGTAGAAG
Deletion compare (Δ**Compare**) (p.Pro408_Gln427del)	c.1221_1280del	CAAGATGAGGAGATGCTTGAAC	ACTGGACAGGGGTTTGCT
**Scramble** p.(M383_D387delinsDTMVR)	c.1147_1160delinsGAC ACTATGGTACG	ggtacgtTCCCGGGAACAGAAGCT T	atagtgtcCTGGTGGGCATAGACCT TATC
**Human-plant ConMot hybrid** p.P379_Y396delinsPVHKMV RTDSLDPAGRLHAY		ctggatccggccggcaggcttcat gcataTCTGCAGCCTCTGAGCAA	ggaatctgtacgaaccatcttgtgg acaggGACCTTATCACTACTTCCAG AAG

Supplementary Table 1: Analyzed variant information.
Variant descriptions confer to HGVS format and refer to the MLH1 reference cDNA (NM_000249.4) or protein (NP_000240.1) sequences and have been checked using Mutalyzer Name Checker.

Supplementary Table 2

	This work		Torres *et al.*	
Organism	Human		Yeast	
Readout	***MMR activity*** (Mismatch repair of a G-T mismatch in a plasmid)		***Endonuclease activation*** (Nicking-induced relaxation of a supercoiled plasmid)	
	Specific (mismatch-induced via MSH2-MSH6)		Non-specific (no mismatch involved)	
Volume		25 µl		40 µl
Proteins	HEK293 nuclear extract	50 µg	Purified components	
	MLH1-PMS2:	220 fmol = 8.7 nM	MLH1-PMS2	35 nM
			PCNA	7.5 nM
			RFC-Δ1N	30 nM
Buffer	Tris-HCl pH 7.5	25 mM	Tris-HCl pH 7.5	20 mM
	Mg^{2+}	5 mM	Mn^{2+}	1 mM
	DTT	5 mM	DTT	2 mM
	ATP	1.5 mM	ATP	0.5 mM
	CTP, GTP, TTP	0.1 mM each	-	-
Peptide	32mer (**activating MMR**): SGSSDKVYAHQ**MVRTD**SREQKLDAFLQPLSK 20mer (**inactive**): KVYAHQ**MVRTD**SREQKLDAF		25mer (inhibiting endonuclease activity): KAKRQENK**LVRID**ASQAKITSFLSS	
	ConMot peptide (molar ratio to hMLH1-PMS2)	0-860-2300-8600	WT peptide (molar ratio to yMLH1-PMS1)	0-250-500-1000

Comparison of reaction conditions in tests with peptides of conserved MLH1 sequences.
The reaction conditions of the peptide supplementation experiments shown in this work are compared to the effects of peptide on yMLH1-yPMS1 endonuclease activation. Differences considered significant are highlighted in red.

Supplementary Figure 1

Overview of conservation of the ConMot motif in different kingdoms of eukaryotes.

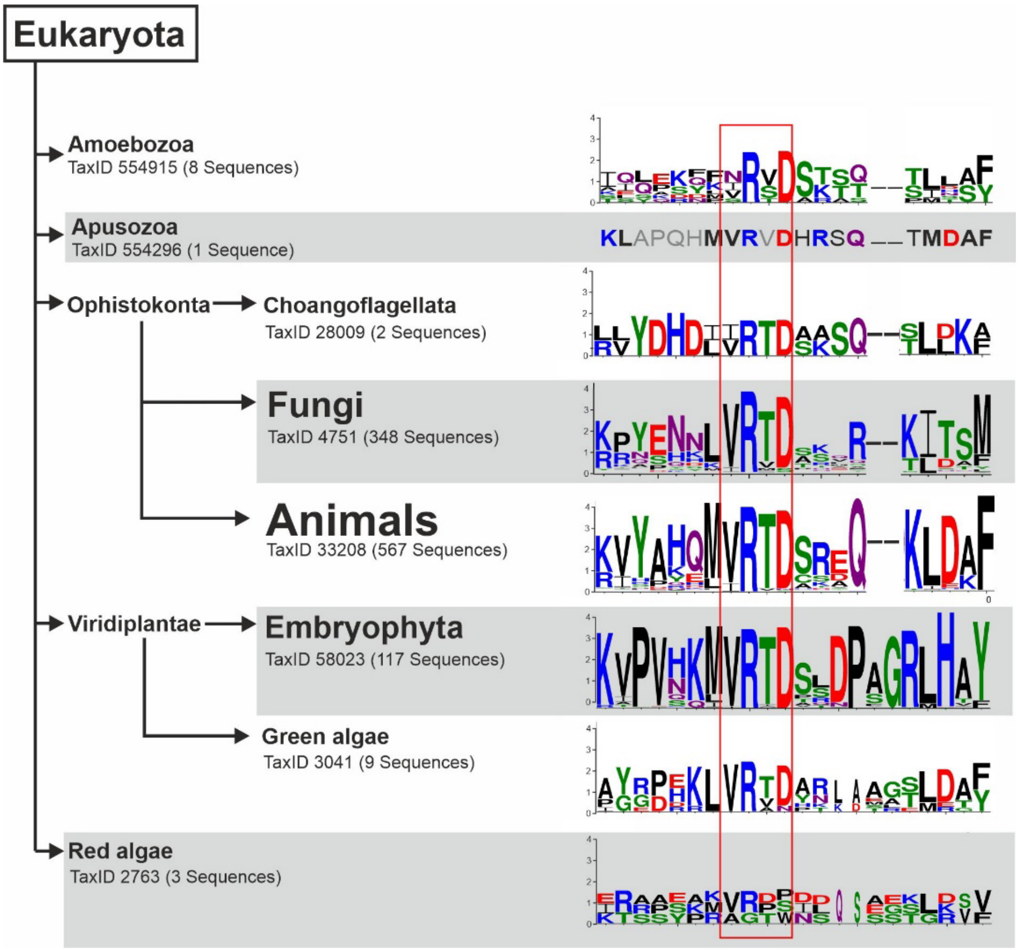

MLH1 sequences of different eukaryotic taxa were retrieved using protein BLAST. Sequences were manually curated by removing incomplete sequences and doublets from one organism. All sequences were confirmed to contain the signature motif of MLH1 proteins (the highly conserved C-terminal FERC motif). Alignments were performed with Muscle. Conservation images of ConMot motifs were created using WebLogo 3.

Supplementary Figure 2

Structure predictions and secondary structure assessment of the ConMot sequence.

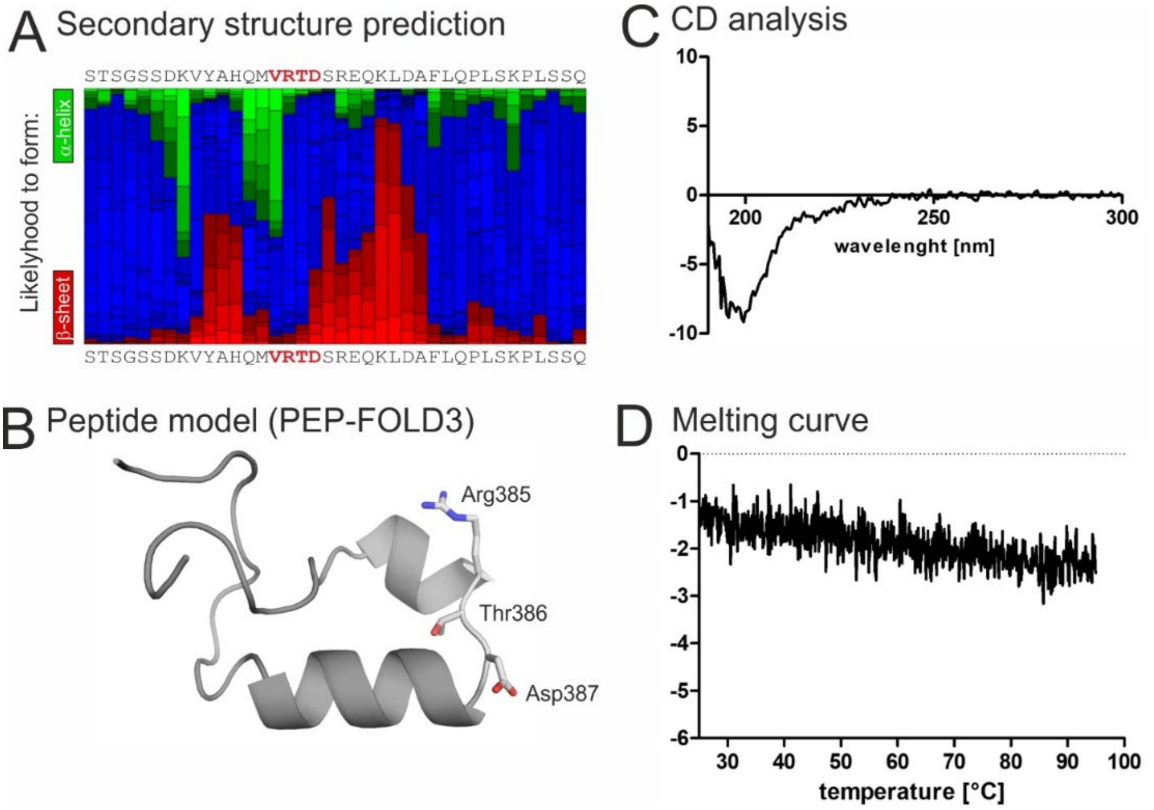

A. PEP-FOLD3 prediction of the propensity of the ConMot to form secondary structures.
B. A folding model for the ConMot peptide base on the PEP-FOLD3 algorithm.
C. CD spectra of a 33µM ConMot peptide sample in 20 mM Na-phosphate buffer pH 7.2 at 20°C.
D. Melting curve (25-95 °C) of the same sample detected at 222 nm.

Supplementary Figure 3

Mismatched DNA plasmid substrate and principle of MMR assay.

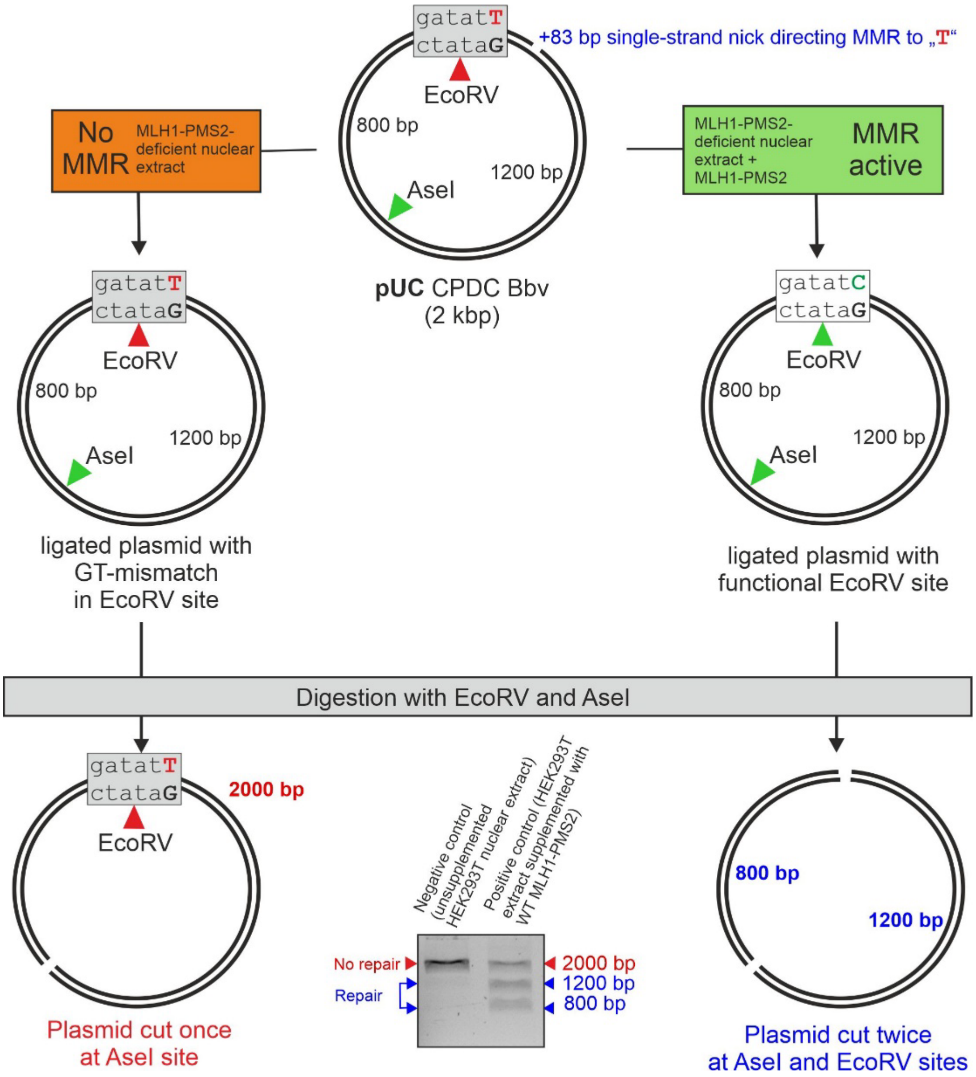

The MMR assay is performed using as substrate a modified 2 kbp derivative of the pUC vector (pUC CPDC Bbv) originally generated by Wang and Hays and further modified by Plotz *et al.* and Gonzáles-Acosta *et al*. (see references in Materials and Methods for comprehensive details on the plasmid and the generation of the substrate). This plasmid is prepared for the assay by introducing a GT-mismatch in an EcoRV restriction site, which is inactive when the mismatch is present. Additionally, a single-strand nick is introduced at a distance of +83 bp in the 3’-direction of the T of the GT-mismatch. When this substrate is incubated with MMR-proficient protein nuclear extract at 37°C for 10-15 minutes, the GT mismatch is repaired to homoduplex GC, making the restriction site competent for digestion by EcoRV. In parallel, the single-strand nick is ligated. This ligation occurs in part independently of the MMR reaction, resulting in a certain fraction of ligated plasmid that has not been repaired by MMR but that is, in the absence of the single-strand nick, no longer accessible for correction by MMR. Therefore, following purification of the plasmid substrate and digestion with EcoRV and AseI, only a part of the plasmid appears repaired (two bands at 1200 bp and 800 bp in an agarose gel) even if MMR was effective. 40-80% absolute repair, determined as signal intensities of the repair bands divided by sum of signal intensities of all plasmid bands, are optimally achieved and allow accurate determination of MMR activity of variants. Without MMR, the EcoRV site is not reconstituted and only the 2000 bp band of the linearized plasmid is detectable after agarose gel electrophoresis. Absolute repair values of tested variants are put in relation to absolute repair values of wildtype (WT) proteins (% of WT) for variant MMR activity characterization.

Supplementary Figure 4

MLH1-PMS2 concentration and ConMot peptide binding dissociation constant.

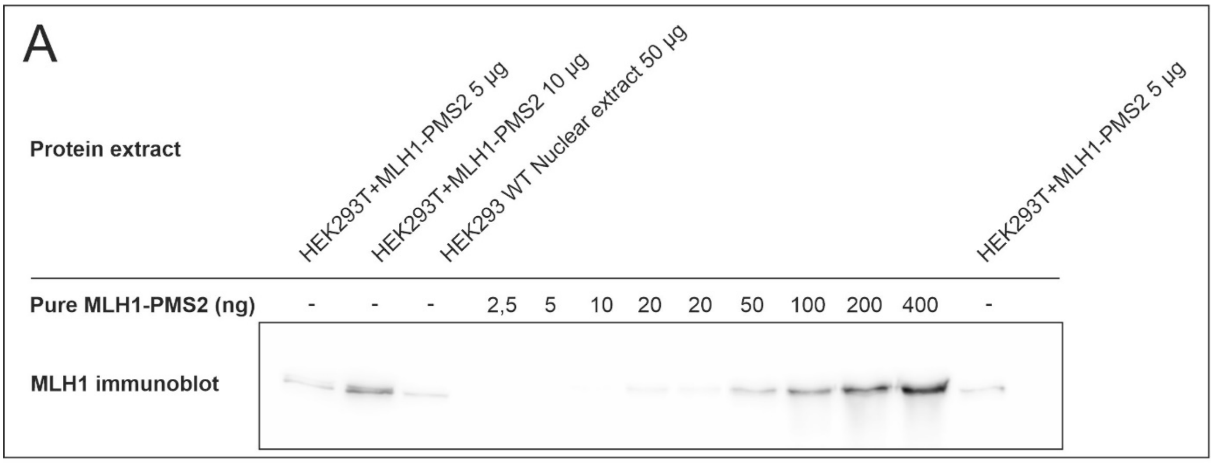

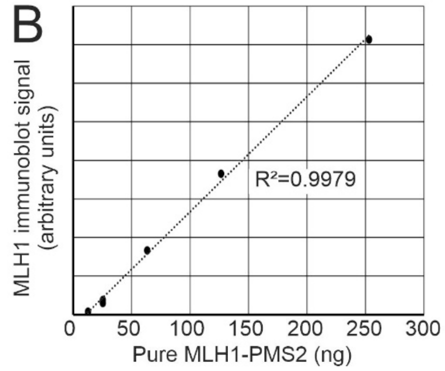

C

i) $K_D = \frac{[A][B]}{[AB]}$

ii) $AB = \frac{(A_T+B_T+K_D)-\sqrt{(A_T+B_T+K_D)^2-4(A_T B_T)}}{2}$

KD:		1	5	10	1	5	10
Protein (MutLα) (=A_T)	Peptide (ConMot) (=B_T)	Protein-peptide complex (=AB)*	Protein-peptide complex (=AB)*	Protein-peptide complex (=AB)*	fraction peptide bound to protein (=AB/A_T)	fraction peptide bound to protein (=AB/A_T)	fraction peptide bound to protein (=AB/A_T)
µM	µM	µM	µM	µM			
0,011123	0,1	0,001002	0,000218	0,000110	0,090	0,020	0,010
0,011123	0,25	0,002209	0,000529	0,000271	0,199	0,048	0,024
0,011123	0,5	0,003689	0,001009	0,000529	0,332	0,091	0,048
0,011123	1	0,005546	0,001851	0,001010	0,499	0,166	0,091
0,011123	2,5	0,007938	0,003704	0,002223	0,714	0,333	0,200
0,011123	7,5	0,009813	0,006671	0,004765	0,882	0,600	0,428
0,011123	18	0,010537	0,008704	0,007149	0,947	0,783	0,643
0,011123	75	0,010977	0,010428	0,009814	0,987	0,937	0,882
0,011123	200	0,011068	0,010852	0,010593	0,995	0,976	0,952
0,011123	1000	0,011112	0,011068	0,011013	0,999	0,995	0,990

A. Protein extract of HEK293T cells transfected with MLH1-PMS2 and purified MLH1-PMS2 in different amounts were separated by SDS-PAGE, and MLH1 was detected by immunoblotting. The applied amount of extract of transfected cells was identical to the amount used in the mismatch repair reactions (5 µg), or double (10 µg). For comparison, 50 µg MLH1-proficient HEK293 nuclear extract was applied.
B. Immunoblot band intensities were determined. There was a good linear correspondence with the applied amount of MLH1-PMS2 (R^2=0.9976). The 400 ng signal was omitted since it was far above the range of interest. The amount of MLH1-PMS2 in cell extracts was determined using the linear regression equation. According to these measurements, the MMR assays performed in this work contained MLH1-PMS2 at a concentration of 11.1 nM.
C. In steady state, the degree of binding of a peptide (B) to a protein (A) depends on the dissociation constant K_D (equation i), which can be rearranged to calculate the concentration of complex using total concentrations in the reaction (A_T and B_T, equation ii). Using this equation, expected concentrations of complex (AB) and of the fractions of peptide bound to protein (AB/A_T) were calculated for three different K_D (1, 5 and 10 µM) (Table) using the total concentration of MLH1 protein A_T determined above (11.1 nM) and a series of concentrations for peptide (BT, ranging from 0.1 to 1000 µM. These values were used for the theoretical binding curves displayed in **Figure 4D**.

Supplementary Figure 5

Estimated quality of the AlphaFold model of the human MLH1-PMS2 C-terminal domains dimer.

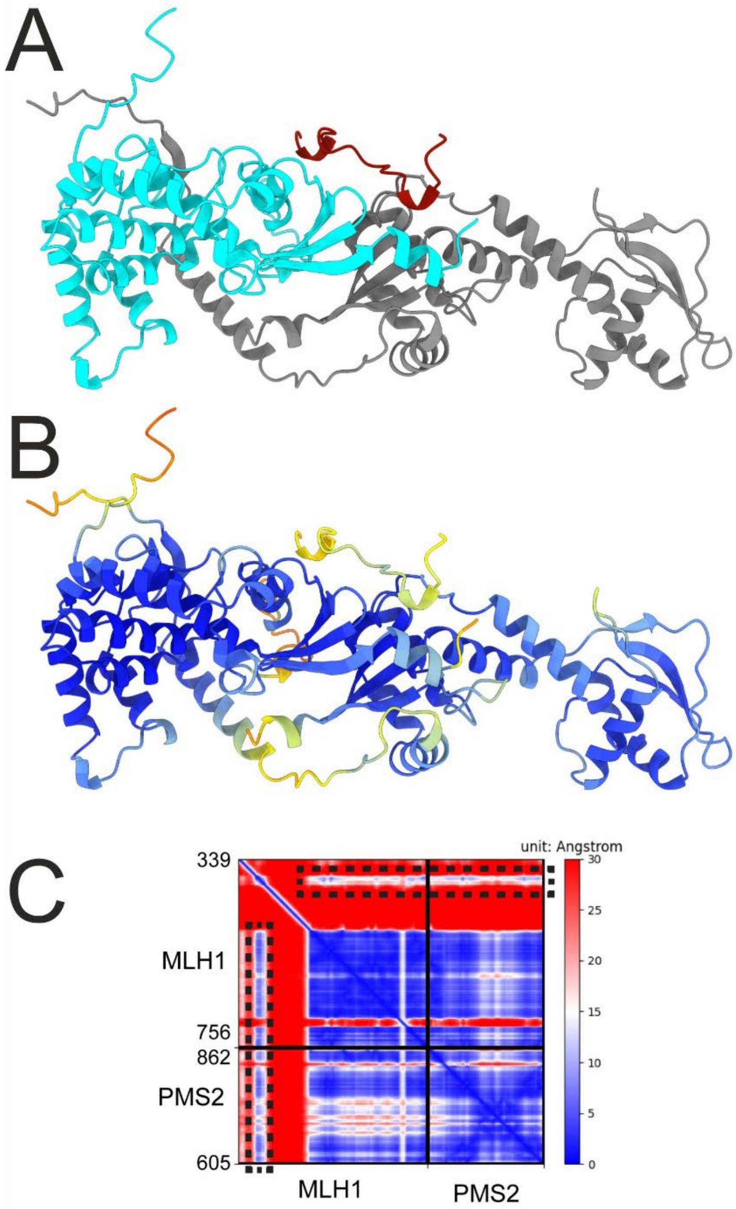

(A) The model of the C-terminal domains of MLH1 (cyan, aa. 339-756) and PMS2 (gray, aa. 605-862). Disordered linker of MLH1 (aa. 339-376 and aa. 397-490) are hidden from view for clarity. ConMot is colored red.

(B) The model colored by local confidence estimated with predicted local distance difference test (pLDDT), as returned by AlphaFold. The pLDDT > 90 (dark blue) indicates high estimated accuracy of backbone and side chain rotamers whereas pLDDT > 70 (light blue) indicates confident backbone prediction.

(C) The confidence of inter- domain and inter-chain orientations estimated with the predicted distance error (PAE) in Å between all pairs of residues in the dimer, as returned by ColabFold. The color at each (x, y) position of the matrix corresponds to the expected distance error in residue x's position, when the prediction and true (unknown) structure are aligned on residue y. Blue indicates low error. The region corresponding to the orientation of ConMot versus MLH1 and PMS2 is indicated with dashed frame. Although the local pLDDT scores of the ConMot region are low, the PAE scores indicate high confidence of the ConMot interaction.

Supplementary Figure 6

Cancer diseases in the p.Arg385Pro variant carrier pedigree.

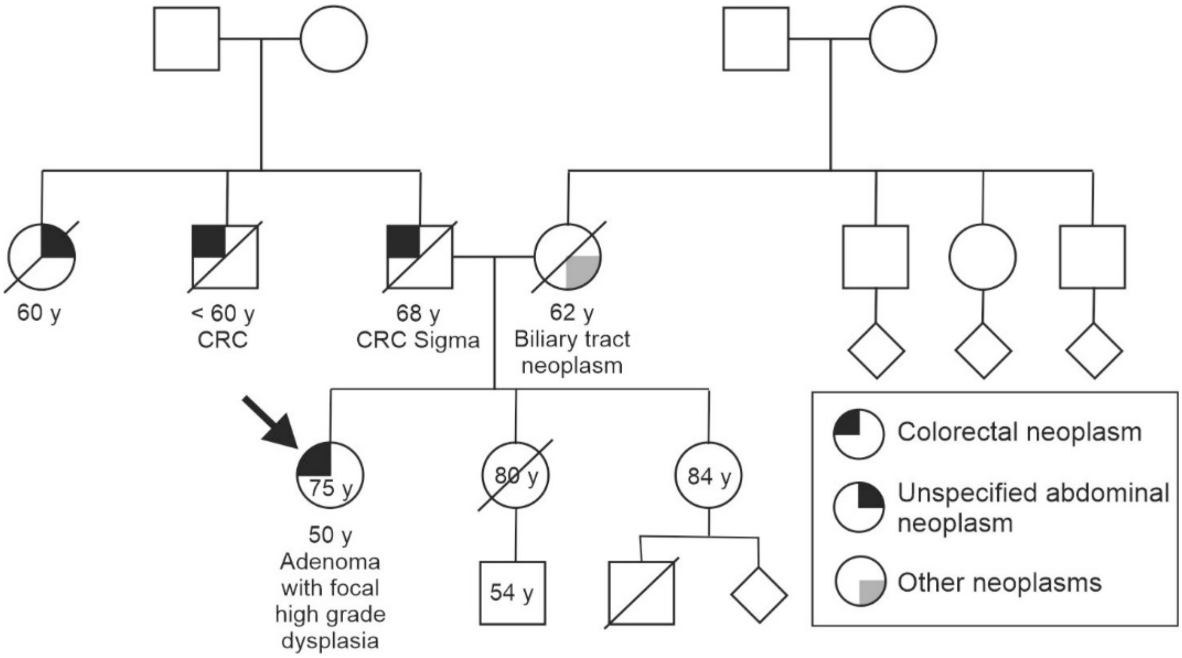

The pedigree of family ID R-RM6 of a patient carrying the MLH1 p.Arg385Pro variant is shown in the top panel. This index patient is marked by an arrow. Squares represent men, circles represent women, a line indicates that the individual is deceased. Black marking signifies tumor entities which are compatible with Lynch syndrome, grey marking other neoplasms. Age at diagnosis in years (y) is given for these individuals. Age within symbols signifies current age (2022), or age at death.
